# Supplementary material for: End Sequence Analysis Toolkit (ESAT) expands the extractable information from single-cell RNA-seq data
Source: Genome Res. 2016 Oct;26(10):1397–410. doi: 10.1101/gr.207902.116 (PMC5052061; doi:10.1101/gr.207902.116)
Supplement: Supplemental Material [file supp_gr.207902.116_Supp_Materials.tar.gz › ESAT-esat_v0.1/doc/html/classumms_1_1core_1_1annotation_1_1_gene-members.html]

ESAT: Member List


ESAT


- Main Page
- Related Pages
- Packages
- Classes
- Files

- Class List
- Class Index
- Class Hierarchy
- Class Members

All Classes Namespaces Files Functions Variables Enumerator Pages

- umms
- core
- annotation
- Gene

umms.core.annotation.Gene Member List

This is the complete list of members for umms.core.annotation.Gene, including all inherited members.

|  |  |  |
| --- | --- | --- |
| absoluteToRelativePosition(int referenceCoordinate) | umms.core.annotation.Gene |  |
| addAttribute(String name, String val) | umms.core.annotation.Gene |  |
| addBlocks(Annotation block) | umms.core.annotation.BasicAnnotation |  |
| addBlocks(Collection<?extends Annotation > blocks) | umms.core.annotation.BasicAnnotation |  |
| addExtraField(String value) | umms.core.annotation.Gene |  |
| addIsoform(Gene g) | umms.core.annotation.Gene |  |
| addSequence(Sequence seq) | umms.core.annotation.Gene |  |
| addSuffixToName(String refName) | umms.core.annotation.Gene |  |
| almostContains(Gene other, int exonFudgeFactor) | umms.core.annotation.Gene |  |
| almostContainsRefSeq(Gene other, int exonFudgeFactor) | umms.core.annotation.Gene | protected |
| almostEqual(Gene other, int exonFudgeFactor) | umms.core.annotation.Gene |  |
| almostSameStructure(Gene other, int intronFudgeFactor) | umms.core.annotation.Gene |  |
| BasicAnnotation() | umms.core.annotation.BasicAnnotation |  |
| BasicAnnotation(SAMRecord samRecord) | umms.core.annotation.BasicAnnotation |  |
| BasicAnnotation(String ucsc) | umms.core.annotation.BasicAnnotation |  |
| BasicAnnotation(String referenceName, int start, int end, Strand orientation, String name) | umms.core.annotation.BasicAnnotation |  |
| BasicAnnotation(String referenceName, Strand orientation, String name, Collection<?extends Annotation > blocks) | umms.core.annotation.BasicAnnotation |  |
| BasicAnnotation(String referenceName, int start, int end) | umms.core.annotation.BasicAnnotation |  |
| BasicAnnotation(String referenceName, int start, int end, Strand orientation) | umms.core.annotation.BasicAnnotation |  |
| BasicAnnotation(String referenceName, int start, int end, String orientation) | umms.core.annotation.BasicAnnotation |  |
| BasicAnnotation(BasicAnnotation other) | umms.core.annotation.BasicAnnotation |  |
| BasicAnnotation(Annotation other) | umms.core.annotation.BasicAnnotation |  |
| BasicAnnotation(Collection<?extends Annotation > blocks) | umms.core.annotation.BasicAnnotation |  |
| BasicAnnotation(Collection<?extends Annotation > blocks, Strand orientation, String name) | umms.core.annotation.BasicAnnotation |  |
| BasicAnnotation(Collection<?extends Annotation > blocks, String name) | umms.core.annotation.BasicAnnotation |  |
| BasicAnnotation(String referenceName, CompoundInterval blocks, Strand orientation, String name) | umms.core.annotation.BasicAnnotation |  |
| BasicAnnotation(String referenceName, CompoundInterval blocks, Strand orientation) | umms.core.annotation.BasicAnnotation |  |
| BasicAnnotation(String referenceName, CompoundInterval blocks) | umms.core.annotation.BasicAnnotation |  |
| blocks | umms.core.annotation.BasicAnnotation | protected |
| compareTo(Annotation other) | umms.core.annotation.Gene |  |
| compareToAnnotation(Annotation b) | umms.core.annotation.AbstractAnnotation |  |
| compareToAnnotation(Annotation b, boolean useOrientation) | umms.core.annotation.AbstractAnnotation |  |
| complement() | umms.core.annotation.BasicAnnotation |  |
| contains(Annotation other) | umms.core.annotation.BasicAnnotation |  |
| containsAttribute(String key) | umms.core.annotation.Gene |  |
| copy() | umms.core.annotation.Gene |  |
| createFromUCSC(String ucsc) | umms.core.annotation.BasicAnnotation | static |
| disect(Annotation a) | umms.core.annotation.BasicAnnotation |  |
| disect(List<?extends Annotation > disectors) | umms.core.annotation.BasicAnnotation |  |
| distanceToNearestNonOverlapper(Collection< Annotation > others) | umms.core.annotation.Gene |  |
| equals(Annotation a) | umms.core.annotation.AbstractAnnotation |  |
| equals(Annotation a, boolean useOrientation) | umms.core.annotation.AbstractAnnotation |  |
| expand(int deltaStart, int deltaEnd) | umms.core.annotation.AbstractAnnotation |  |
| expandUtrs(Integer utr1, Integer utr2) | umms.core.annotation.Gene |  |
| extendAnnotation(int deltaStart, int deltaEnd) | umms.core.annotation.Gene |  |
| findAllORFs(boolean trim3UTRs) | umms.core.annotation.Gene |  |
| findAllORFs(String sequence) | umms.core.annotation.Gene | static |
| findAllORFs(Sequence chrSeq, boolean trim3UTRs) | umms.core.annotation.Gene |  |
| findAllStartCodons() | umms.core.annotation.Gene |  |
| findLongestORF() | umms.core.annotation.Gene |  |
| findLongestORF(String sequence) | umms.core.annotation.Gene | static |
| fromFullInfoString(String fullInfoString) | umms.core.annotation.BasicAnnotation | static |
| fullyContains(Annotation other) | umms.core.annotation.AbstractAnnotation |  |
| Gene(String chr, String name, Strand orientation, Collection<?extends Annotation > exons, int cdsStart, int cdsEnd) | umms.core.annotation.Gene |  |
| Gene(String chr, int start, int end, String name, Strand orientation, int cdsStart, int cdsEnd) | umms.core.annotation.Gene |  |
| Gene(Collection<?extends Annotation > exons, String name, Strand orientation, int cdsStart, int cdsEnd) | umms.core.annotation.Gene |  |
| Gene(String chr, String name, Strand orientation, Collection<?extends Annotation > exons) | umms.core.annotation.Gene |  |
| Gene(Annotation annotation, String name, Strand orientation, Collection<?extends Annotation > exons) | umms.core.annotation.Gene |  |
| Gene(Annotation annotation, Collection<?extends Annotation > exons) | umms.core.annotation.Gene |  |
| Gene(Annotation annotation, Collection<?extends Annotation > exons, String name, int cdsStart, int cdsEnd) | umms.core.annotation.Gene |  |
| Gene(Annotation annotation, Collection<?extends Annotation > exons, String name) | umms.core.annotation.Gene |  |
| Gene(Collection<?extends Annotation > exons, String name, Strand orientation) | umms.core.annotation.Gene |  |
| Gene(String chr, int start, int end, String name, Strand orientation) | umms.core.annotation.Gene |  |
| Gene(String chr, int start, int end, String name) | umms.core.annotation.Gene |  |
| Gene(Collection<?extends Annotation > exons, String name) | umms.core.annotation.Gene |  |
| Gene(Collection<?extends Annotation > exons) | umms.core.annotation.Gene |  |
| Gene(String chr, int start, int end) | umms.core.annotation.Gene |  |
| Gene(Alignments align) | umms.core.annotation.Gene |  |
| Gene(Annotation annotation) | umms.core.annotation.Gene |  |
| Gene(String pslString) | umms.core.annotation.Gene |  |
| Gene(String pslString, boolean isPSLFormat) | umms.core.annotation.Gene |  |
| Gene(String chr2, int start2, int end, String name2, double bedScore2, String orientation2, String sequence2, String[] extraFields2) | umms.core.annotation.Gene |  |
| Gene(String chr2, int start2, int end, String name2, double bedScore2, String orientation2, String sequence2) | umms.core.annotation.Gene |  |
| Gene(String chr, int start, int end, String name, double mybedScore, String orientation, int[] exonsStart, int[] exonsEnd) | umms.core.annotation.Gene |  |
| Gene(String chr, int start, int end, String name, String orientation, List< Integer > exonsStart, List< Integer > exonsEnd) | umms.core.annotation.Gene |  |
| Gene(String chr, int start, int end, String name, String orientation, List< Integer > exonsStart, List< Integer > exonsEnd, int blockStart, int blockEnd) | umms.core.annotation.Gene |  |
| Gene(String chr, int start, int end, String name, String orientation, List< Integer > exonsStart, List< Integer > exonsEnd, String[] extraData) | umms.core.annotation.Gene |  |
| Gene(String chr2, int start2, int end, String name2, double bedScore2, String orientation2, int[] exonStarts2, int[] exonEnds2, double[] exonScores2, String sequence2) | umms.core.annotation.Gene |  |
| Gene(String chr2, int start2, int end, String name2, double bedScore2, String orientation2, int[] exonStarts2, int[] exonEnds2, double[] exonScores2, String sequence2, String[] extraFields2) | umms.core.annotation.Gene |  |
| Gene(String chr, int start, int end, String name, double score, String strand, List< Integer > exonsStart, List< Integer > exonsEnd, int blockStart2, int blockEnd2) | umms.core.annotation.Gene |  |
| Gene(String chr, int start, int end, String name, String orientation, List< Integer > exonsStart, List< Integer > exonsEnd, int blockStart, int blockEnd, String[] extraData) | umms.core.annotation.Gene |  |
| Gene(String chr, int start, int end, String name, double score, String strand, List< Integer > exonsStart, List< Integer > exonsEnd, int blockStart2, int blockEnd2, String[] extraColumns) | umms.core.annotation.Gene |  |
| Gene(Gene gene) | umms.core.annotation.Gene |  |
| geneSpanContains(Gene gene) | umms.core.annotation.Gene |  |
| genomicToGenomicPositionWithOffset(int genomicPosition, int offset) | umms.core.annotation.Gene |  |
| genomicToTranscriptPosition(int genomicPosition) | umms.core.annotation.Gene |  |
| get3PrimeExon(int size) | umms.core.annotation.Gene |  |
| get3PrimeExon() | umms.core.annotation.Gene |  |
| get3PrimeGenomicDistance(LightweightGenomicAnnotation annot) | umms.core.annotation.Gene |  |
| get3Utr() | umms.core.annotation.Gene |  |
| get3UTR() | umms.core.annotation.Gene |  |
| get3UTRGene() | umms.core.annotation.Gene |  |
| get3UtrIncludingIntrons() | umms.core.annotation.Gene |  |
| get5PrimeExon() | umms.core.annotation.Gene |  |
| get5Utr() | umms.core.annotation.Gene |  |
| get5UTR() | umms.core.annotation.Gene |  |
| get5UTRGene() | umms.core.annotation.Gene |  |
| get5UtrIncludingIntrons() | umms.core.annotation.Gene |  |
| getAlignment() | umms.core.annotation.Gene |  |
| getAllPositions() | umms.core.annotation.Gene |  |
| getAttribute(String name) | umms.core.annotation.Gene |  |
| getAttributes() | umms.core.annotation.Gene |  |
| getBedScore() | umms.core.annotation.Gene |  |
| getBlocks() | umms.core.annotation.BasicAnnotation |  |
| getBlocks(boolean oriented) | umms.core.annotation.BasicAnnotation |  |
| getCDS() | umms.core.annotation.Gene |  |
| getCDSEnd() | umms.core.annotation.Gene |  |
| getCDSRegion() | umms.core.annotation.Gene |  |
| getCDSStart() | umms.core.annotation.Gene |  |
| getChr() | umms.core.annotation.AbstractAnnotation |  |
| getChrNum() | umms.core.annotation.Gene |  |
| getCountScore() | umms.core.annotation.Gene |  |
| getDistanceTo(Annotation other) | umms.core.annotation.AbstractAnnotation |  |
| getEnd() | umms.core.annotation.BasicAnnotation |  |
| getExons() | umms.core.annotation.Gene |  |
| getExonSet() | umms.core.annotation.Gene |  |
| getExonsScores() | umms.core.annotation.Gene |  |
| getExonTree() | umms.core.annotation.Gene |  |
| getExtraFields() | umms.core.annotation.Gene |  |
| getExtraFields(int i) | umms.core.annotation.Gene |  |
| getFirstExon() | umms.core.annotation.Gene |  |
| getFirstIntron() | umms.core.annotation.Gene |  |
| getFlankingBlocks(Annotation spliceJunction) | umms.core.annotation.BasicAnnotation |  |
| getFullInfoString() | umms.core.annotation.AbstractAnnotation |  |
| getGappedSize() | umms.core.annotation.Gene |  |
| getGenomicLength() | umms.core.annotation.Gene |  |
| getIntrons() | umms.core.annotation.Gene |  |
| getIntronsBlocks() | umms.core.annotation.Gene |  |
| getIntronSet() | umms.core.annotation.Gene |  |
| getIntronTranscript() | umms.core.annotation.Gene |  |
| getIsoforms() | umms.core.annotation.Gene |  |
| getLastExon() | umms.core.annotation.Gene |  |
| getLastIntron() | umms.core.annotation.Gene |  |
| getLengthOnReference() | umms.core.annotation.AbstractAnnotation |  |
| getMidpoint() | umms.core.annotation.BasicAnnotation |  |
| getMidpointGenomicCoords() | umms.core.annotation.Gene |  |
| getName() | umms.core.annotation.BasicAnnotation |  |
| getNormalizedScore() | umms.core.annotation.Gene |  |
| getNumberPositions(int rate) | umms.core.annotation.Gene |  |
| getNumExons() | umms.core.annotation.Gene |  |
| getOrientation() | umms.core.annotation.BasicAnnotation |  |
| getOrientedEnd() | umms.core.annotation.AbstractAnnotation |  |
| getOrientedLastExon() | umms.core.annotation.Gene |  |
| getOrientedStart() | umms.core.annotation.AbstractAnnotation |  |
| getOverlap(Gene second) | umms.core.annotation.Gene |  |
| getOverlap(Collection< Gene > others) | umms.core.annotation.Gene |  |
| umms::core::annotation::BasicAnnotation.getOverlap(Annotation other) | umms.core.annotation.BasicAnnotation |  |
| getOverlappers(Collection< Gene > genes, String chr, int start, int end, Strand strand, boolean ignoreOrientation) | umms.core.annotation.Gene | static |
| getPercent3UTR() | umms.core.annotation.Gene |  |
| getPercentCDS() | umms.core.annotation.Gene |  |
| getPositionAtReferenceCoordinate(int referenceCoordinate, boolean ignoreOrientation) | umms.core.annotation.BasicAnnotation |  |
| umms::core::annotation::AbstractAnnotation.getPositionAtReferenceCoordinate(int referenceCoordinate) | umms.core.annotation.AbstractAnnotation |  |
| getPromoter(int fudgeFactor) | umms.core.annotation.Gene |  |
| getPromoter(int upstream, int downstream) | umms.core.annotation.Gene |  |
| getPValue() | umms.core.annotation.Gene |  |
| getReferenceCoordinateAtPosition(int positionInAnnotation, boolean ignoreOrientation) | umms.core.annotation.BasicAnnotation |  |
| umms::core::annotation::AbstractAnnotation.getReferenceCoordinateAtPosition(int positionInAnnotation) | umms.core.annotation.AbstractAnnotation |  |
| getReferenceName() | umms.core.annotation.BasicAnnotation |  |
| getRPKM() | umms.core.annotation.Gene |  |
| getSAMEnd() | umms.core.annotation.AbstractAnnotation |  |
| getSAMStart() | umms.core.annotation.AbstractAnnotation |  |
| getSAMString() | umms.core.annotation.Gene |  |
| getScore() | umms.core.annotation.BasicAnnotation |  |
| getSequence() | umms.core.annotation.Gene |  |
| getSequence(Sequence chrSequence) | umms.core.annotation.Gene |  |
| getSequence(Sequence chr2, boolean repeatMask, boolean stranded) | umms.core.annotation.Gene |  |
| getSequenceObject() | umms.core.annotation.Gene |  |
| getSize() | umms.core.annotation.Gene |  |
| getSortedAndUniqueExons() | umms.core.annotation.Gene |  |
| getSpliceConnections() | umms.core.annotation.BasicAnnotation |  |
| getSpliceJunctionCoordinates() | umms.core.annotation.Gene |  |
| getSpliceJunctionCoordinatesTree() | umms.core.annotation.Gene |  |
| getStart() | umms.core.annotation.BasicAnnotation |  |
| getStartCodon() | umms.core.annotation.Gene |  |
| getStrand() | umms.core.annotation.AbstractAnnotation |  |
| getStrand(String orientation) | umms.core.annotation.AbstractAnnotation | static |
| getStrand(char orientation) | umms.core.annotation.AbstractAnnotation | static |
| getString() | umms.core.annotation.Gene |  |
| getTranscriptLength() | umms.core.annotation.Gene |  |
| getWindows(int windowSize) | umms.core.annotation.Gene |  |
| getWindows(int windowSize, int stepSize, int start) | umms.core.annotation.Gene |  |
| hasCDS() | umms.core.annotation.Gene |  |
| hashCode() | umms.core.annotation.Gene |  |
| hasOrientation() | umms.core.annotation.AbstractAnnotation |  |
| initFromGene(Gene gene) | umms.core.annotation.Gene | protected |
| intersect(Annotation other) | umms.core.annotation.BasicAnnotation |  |
| umms::core::annotation::AbstractAnnotation.intersect(List<?extends Annotation > others) | umms.core.annotation.AbstractAnnotation |  |
| isCodingGene() | umms.core.annotation.Gene |  |
| isFullyCompatible(Gene iso) | umms.core.annotation.Gene |  |
| isInExon(int snp) | umms.core.annotation.Gene |  |
| isNegativeStrand() | umms.core.annotation.Gene |  |
| isStopCodon(String currentCodon) | umms.core.annotation.Gene | static |
| isUnoriented() | umms.core.annotation.BasicAnnotation |  |
| length() | umms.core.annotation.BasicAnnotation |  |
| main(String[] args) | umms.core.annotation.BasicAnnotation | static |
| MAX\_DISTANCE | umms.core.annotation.AbstractAnnotation | static |
| minus(Annotation other) | umms.core.annotation.BasicAnnotation |  |
| minus(Collection<?extends Annotation > others) | umms.core.annotation.BasicAnnotation |  |
| moveToCoordinate(int coordinateInReference) | umms.core.annotation.BasicAnnotation |  |
| numBlocks() | umms.core.annotation.BasicAnnotation |  |
| numOfCompatibleIntrons(Gene g) | umms.core.annotation.Gene |  |
| numOverlappingExons(Gene iso) | umms.core.annotation.Gene |  |
| overlaps(Gene other) | umms.core.annotation.Gene |  |
| overlaps(Gene other, boolean ignoreOrientation) | umms.core.annotation.Gene |  |
| overlaps(Gene other, double minPctOverlap) | umms.core.annotation.Gene |  |
| umms::core::annotation::BasicAnnotation.overlaps(Annotation other, int buffer, boolean considerOrientation) | umms.core.annotation.BasicAnnotation |  |
| umms::core::annotation::BasicAnnotation.overlaps(List<?extends Annotation > otherBlocks, int buffer) | umms.core.annotation.BasicAnnotation |  |
| umms::core::annotation::AbstractAnnotation.overlaps(Annotation other) | umms.core.annotation.AbstractAnnotation |  |
| umms::core::annotation::AbstractAnnotation.overlaps(Annotation other, int buffer) | umms.core.annotation.AbstractAnnotation |  |
| umms::core::annotation::AbstractAnnotation.overlaps(Annotation other, boolean considerOrientation) | umms.core.annotation.AbstractAnnotation |  |
| umms::core::annotation::AbstractAnnotation.overlaps(Collection<?extends Annotation > others) | umms.core.annotation.AbstractAnnotation |  |
| umms::core::annotation::AbstractAnnotation.overlaps(Collection<?extends Annotation > others, int buffer) | umms.core.annotation.AbstractAnnotation |  |
| overlaps2(LightweightGenomicAnnotation next) | umms.core.annotation.Gene |  |
| overlapsExon(Annotation gen) | umms.core.annotation.Gene |  |
| overlapsExon(LightweightGenomicAnnotation exon) | umms.core.annotation.Gene |  |
| overlapsGene(Collection< Gene > others) | umms.core.annotation.Gene |  |
| overlapsGene(Gene gen) | umms.core.annotation.Gene |  |
| overlapsGeneInAnyOrientation(Gene otherGene) | umms.core.annotation.Gene |  |
| overlapsStranded(Gene other, double minPctOverlap) | umms.core.annotation.Gene |  |
| umms::core::annotation::BasicAnnotation.overlapsStranded(Annotation other) | umms.core.annotation.AbstractAnnotation |  |
| percentGenomeOverlapping(Gene gene2) | umms.core.annotation.Gene |  |
| percentOverlapping(Gene gene2) | umms.core.annotation.Gene |  |
| setBedScore(double d) | umms.core.annotation.Gene |  |
| setCDS(Annotation cdsRegion) | umms.core.annotation.Gene |  |
| setCDSEnd(int end) | umms.core.annotation.Gene |  |
| setCDSRegion(int start, int end) | umms.core.annotation.Gene |  |
| setCDSStart(int start) | umms.core.annotation.Gene |  |
| setCountScore(double score) | umms.core.annotation.Gene |  |
| setEnd(int end) | umms.core.annotation.BasicAnnotation |  |
| setExtraFields(double[] d) | umms.core.annotation.Gene |  |
| setExtraFields(double val, int i) | umms.core.annotation.Gene |  |
| setExtraFields(String[] extraData) | umms.core.annotation.Gene |  |
| setName(String n) | umms.core.annotation.BasicAnnotation |  |
| setOrientation(String orientation) | umms.core.annotation.Gene |  |
| umms::core::annotation::BasicAnnotation.setOrientation(Strand orientation) | umms.core.annotation.BasicAnnotation |  |
| umms::core::annotation::AbstractAnnotation.setOrientation(char orientation) | umms.core.annotation.AbstractAnnotation |  |
| setOrientedEnd(int orientedEnd) | umms.core.annotation.BasicAnnotation |  |
| setOrientedStart(int orientedStart) | umms.core.annotation.BasicAnnotation |  |
| setReferenceName(String refName) | umms.core.annotation.BasicAnnotation |  |
| setSAMString(String sam) | umms.core.annotation.Gene |  |
| setScore(double s) | umms.core.annotation.BasicAnnotation |  |
| setSequence(String seq) | umms.core.annotation.Gene |  |
| setSequenceFromChromosome(Sequence chrSequence) | umms.core.annotation.Gene |  |
| setStart(int start) | umms.core.annotation.BasicAnnotation |  |
| shift(int delta) | umms.core.annotation.BasicAnnotation |  |
| size() | umms.core.annotation.AbstractAnnotation |  |
| START\_CODON\_PATRN | umms.core.annotation.Gene | static |
| stitchTo(Annotation next) | umms.core.annotation.BasicAnnotation |  |
| STOP\_CODONS | umms.core.annotation.Gene | static |
| takeUnion(Gene other) | umms.core.annotation.Gene |  |
| toBED() | umms.core.annotation.Gene |  |
| toBED(int r, int g, int b) | umms.core.annotation.Gene |  |
| toBED(boolean useExtraFields) | umms.core.annotation.Gene |  |
| toBED(boolean useExtraFields, int r, int g, int b) | umms.core.annotation.Gene |  |
| toBEDGraph() | umms.core.annotation.AbstractAnnotation |  |
| toBEDwithBedScore() | umms.core.annotation.Gene |  |
| toCigar() | umms.core.annotation.AbstractAnnotation |  |
| toCufflinksGTF(String source, String geneName, String transcriptName, String nonParsedAttrs) | umms.core.annotation.Gene |  |
| toGTF(String source) | umms.core.annotation.Gene |  |
| toGTF(String source, String geneName, String transcriptName) | umms.core.annotation.Gene |  |
| toRefSeq() | umms.core.annotation.Gene |  |
| toSAM() | umms.core.annotation.Gene |  |
| toSAM(String originalSAMLine) | umms.core.annotation.Gene |  |
| toSAM(String originalSAMLine, boolean flip) | umms.core.annotation.Gene |  |
| toShortBED() | umms.core.annotation.AbstractAnnotation |  |
| toString() | umms.core.annotation.Gene |  |
| toUCSC() | umms.core.annotation.Gene |  |
| transcriptToGenomicPosition(int transcriptPosition) | umms.core.annotation.Gene |  |
| transcriptToGenomicPosition(int startPosOnTranscript, int endPosOnTranscript) | umms.core.annotation.Gene |  |
| trim(int deltaStart, int deltaEnd) | umms.core.annotation.AbstractAnnotation |  |
| trimAbsolute(int alignmentStart, int alignmentEnd) | umms.core.annotation.Gene |  |
| trimGene(int relativeStart, int relativeEnd) | umms.core.annotation.Gene |  |
| union(Annotation other) | umms.core.annotation.BasicAnnotation |  |


---

Generated on Fri Aug 15 2014 15:11:09 for ESAT by  

 1.8.7
